# Basal ganglia for beginners: the basic concepts you need to know and their role in movement control

**DOI:** 10.3389/fnsys.2023.1242929

**Published:** 2023-08-03

**Authors:** Gabriel S. Rocha, Marco A. M. Freire, André M. Britto, Karina M. Paiva, Rodrigo F. Oliveira, Ivana A. T. Fonseca, Dayane P. Araújo, Lucidio C. Oliveira, Fausto P. Guzen, Paulo L. A. G. Morais, José R. L. P. Cavalcanti

**Affiliations:** Laboratory of Experimental Neurology, Department of Biomedical Sciences, State University of Rio Grande do Norte, Mossoró, Brazil

**Keywords:** basal ganglia, substantia nigra, striatum, dopamine, movement control, movement disorders

## Abstract

The basal ganglia are a subcortical collection of interacting clusters of cell bodies, and are involved in reward, emotional, and motor circuits. Within all the brain processing necessary to carry out voluntary movement, the basal nuclei are fundamental, as they modulate the activity of the motor regions of the cortex. Despite being much studied, the motor circuit of the basal ganglia is still difficult to understand for many people at all, especially undergraduate and graduate students. This review article seeks to bring the functioning of this circuit with a simple and objective approach, exploring the functional anatomy, neurochemistry, neuronal pathways, related diseases, and interactions with other brain regions to coordinate voluntary movement.

## 1. Basic concepts about movement

Movement is an essential ability to maintain life. Even fewer complex beings such as bacteria need to move in search of nutrients and ensure their survival (Lodish et al., 2021). In humans, movement goes beyond locomotion and involves a myriad of important aspects, such as object manipulation, verbal communication, feeding and vision, since we need eye movements to search for and maintain in visual focus anything that is the target of our attention (Bear et al., 2020). Voluntary movement is prepared and initiated in the motor cortex; such preparation mainly involves corticothalamic projections, while the initiation of motor action consists of corticospinal projections (Economo et al., 2018). The axons of neurons originated in the motor cortex travel through the spinal cord and perform synapses in motor neurons that innervate muscle tissue, so that the desired movement is achieved (Jackson, 2018).

Other brain regions, such as cerebellum and basal ganglia, act together with the motor cortex to provide precise movements. These two structures modulate motor actions through neuronal circuits known as motion adjustment loops, as they regulate movement by adjusting the activity of upper motor neurons, and do not perform direct synapse with lower motor neurons (Purves et al., 2017). The cerebellum, despite having a considerably smaller size than the cortex, has a large neuronal density, with about 69 of the 86 billion nervous cells that make up the encephalon, which corresponds to approximately 80% of the total amount (Azevedo et al., 2009). Considering the motricity, this immense density in the cerebellum is linked to its involvement in various afferent and efferent pathways. It receives information about the body position and movement through the spinal cord and sends it to the motor cortex and the descending motor system, therefore responsible for maintaining posture, balance, movement correction and motor learning (Damiani et al., 2016). For such reasons, the cerebellum is the encephalic region where the movement planned is compared with the one executed, thus providing feedback so that the individual can succeed in his future motor actions, if the previous movement has not been correct (Bear et al., 2020).

The basal ganglia are a subcortical cluster of interconnected cell bodies, which interact with each other through circuits that mainly modulate motricity (Parent, 2012), but are also involved in coordination of behavioral and emotional functions (Lanciego et al., 2012). Due to its considerable contribution to the motor control pathways, some movement disorders, such as Parkinson’s disease, dystonia’s and dyskinesias are caused by disturbances in the basal ganglia (Wichmann and Dostrovsky, 2011). Although widely studied, the motor pathways of the basal ganglia are seen as complex and difficult to understand for didactic purposes. Therefore, the aim of this review is to be a simplified and straightforward version for undergraduate and graduate students who wish to have a very first contact with the basal ganglia topic and its role in movement control, describing the functional anatomy and neurochemical factors involved.

## 2. Functional anatomy of the basal ganglia

**FIGURE 1:**
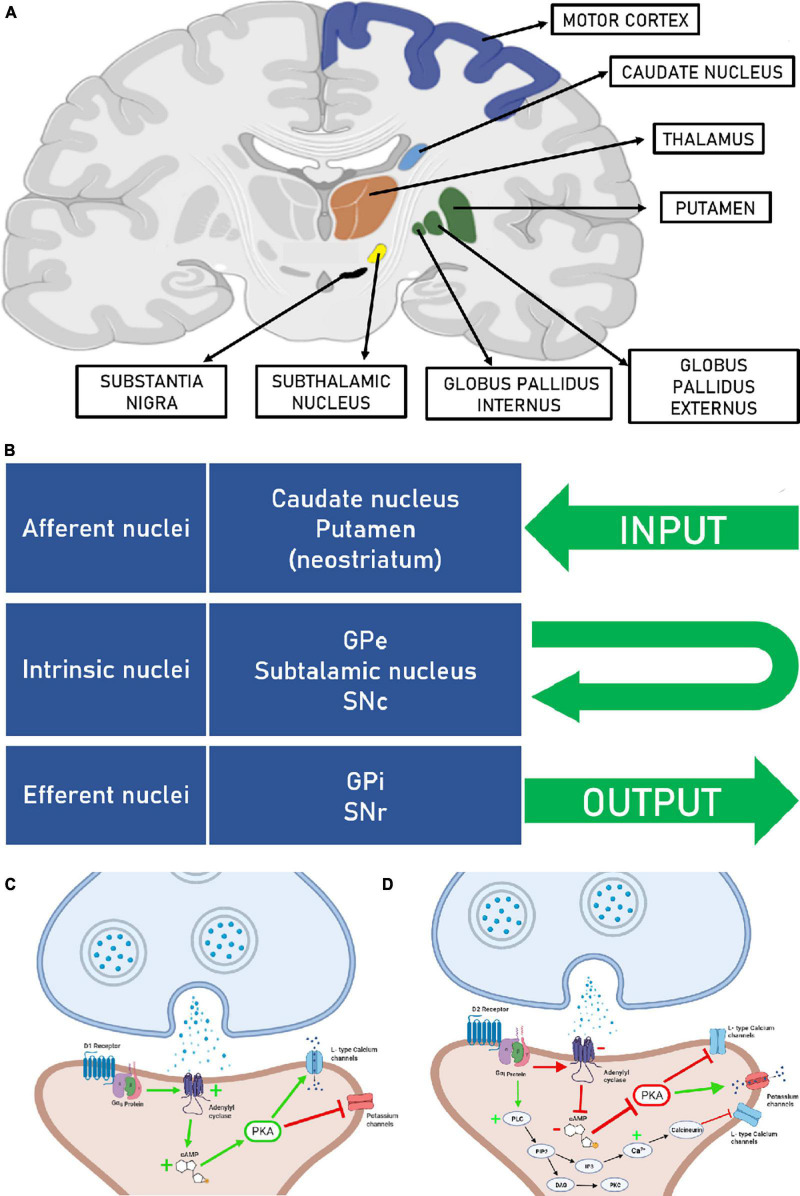
**(A)** Coronal section of the brain, showing the structures that are part of the basal ganglia and the motor cortex (Created at biorender.com). **(B)** Afferent, intrinsic and efferent nuclei of the basal ganglia. Globus pallidus externus (GPe), globus pallidus internus (GPi), substantia nigra pars compacta (SNc), and substantia nigra pars reticulata (SNr). **(C)** Right after dopamine binds to the D1 receptor, the α subunit of the Gs protein moves to adenylate cyclase, stimulating this enzyme to produce more cAMP. Increased levels of cAMP lead to activation of PKA, which consequently phosphorylate L-type calcium channels and potassium channels, phosphorylation activates calcium channels and inhibits potassium channels, making the neuron more susceptible to depolarization. **(D)** In the D2 receptor activation, the α subunit of the Gi protein moves to adenylate cyclase, inhibiting this enzyme to produce cAMP. Low levels of cAMP make the PKA inactivated, which consequently does not phosphorylate L-type calcium channels and potassium channels, making the neuron less susceptible to depolarization. It is also possible to see a second pathway activated by the D2 receptor. PLC catalyzes the hydrolysis of PIP2 to DAG and IP3, DAG activates PKC, and IP3 generates increased cytosolic Ca^2+^ by binding to receptors on the endoplasmic reticulum membrane. The transient increase in Ca^2+^ causes activation of calcineurin, which in turn activates other protein kinases that suppress the activity of L-type Ca^2+^ channels.

According to Steiner and Tseng (2016), the canonical structures that integrate the basal ganglia are: striatum, that in primates are subdivided into three structures known as caudate nucleus, putamen, that form the dorsal region of striatum and nucleus accumbens, which corresponds to the ventral part of the striatum. The other parts are the globus pallidus externus (GPe), globus pallidus internus (GPi), subthalamic nucleus (STN), which is part of the diencephalon and substantia nigra (SN), which is divided into pars compacta (SNc) and pars reticulata (SNr) that are part of the midbrain (Figure 1A). Regarding the projections, the basal ganglia are divided into afferent, efferent and intrinsic nuclei. The afferent nuclei are regions where the information entries. The caudate nucleus and putamen receive corticostriatal inputs related to the motricity pathway and the nucleus accumbens from the emotional and reward pathways (Martin, 2012). The efferent nuclei are responsible for the output to other brain regions and are included the GPi and SNr. Intrinsic nuclei are involved in circuits between the components of the basal ganglia, and it is composed of GPe, STN and SNc (Lanciego et al., 2012; Figure 1B). To understand how these nuclei work together it is important to understand each one of them individually.

### 2.1. Afferent nuclei

Striatum is composed mostly of GABAergic projection neurons (γ-aminobutyric acid), known as medium spiny neurons, due to the large number of spines in their dendrites. These cells also produce and release some neuropeptides such as enkephalin, substance P, and dynorphin (Fazl and Fleisher, 2018), which amplify GABA’s inhibitory action. In less amount, there are interneurons in the striatum that have as a neurotransmitter GABA, acetylcholine, somatostatin, nitric oxide, neuropeptide Y. Striatal interneurons act mainly by modulating the medium spiny neurons, defining whether or not they will be activated (Kawaguchi, 1997; Assous and Tepper, 2019).

The striatum is the main region for inputs to the basal ganglia, receiving several projections from the cortex, thalamus, and brainstem. The cortico-striatal projections exert the greatest influence on the striatum through glutamatergic synapses, they are mostly excitatory. Another structure that contributes with glutamatergic inputs to the striatum is the thalamus, however, it does not impose the same impact on striatal activity as the cortical afferences. The pedunculopontine nucleus and dorsolateral pontine tegmentum are structures in the brainstem and perform cholinergic projections for both medium spiny neurons and striatal interneurons, nonetheless their functions are not yet fully understood so far. In addition, the midbrain sends large-scale dopaminergic outputs to striatum (Silberberg and Bolam, 2015). Dopaminergic signaling is fundamental for the basal ganglia’s motor circuit. As such, among midbrain dopaminergic nuclei, the SN plays a key role in this pathway, because one of its subdivisions, the SNc, communicates directly with the striatum in a pathway known as nigrostriatal pathway (Rice et al., 2011; Norrara et al., 2018; Rocha et al., 2022).

Once it receives dopaminergic inputs in large amounts, the striatum has high density of dopamine receptors. Dopamine receptors are divided into D_1_ and D_2_ types, which are metabotropic and exert modulatory effects on neuron activity. Both receptor types are coupled to G proteins (GPCRs) and create responses through a cascade of intracellular signaling. It activates or inhibits key proteins in specific pathways, influencing the neuron depolarization rate (Tritsch and Sabatini, 2012; Steiner and Tseng, 2016).

D_1_ like receptors are coupled with the G_α*s*_ protein. When D_1_ agonists attach, the α subunit displacement from protein G to the enzyme adenylyl cyclase, causing its activation. Then it catalyzes the conversion of adenosine triphosphate (ATP) into cyclic adenosine monophosphate (cAMP). The increased intracellular concentration of cAMP results in kinase A protein (PKA) activation, which phosphorylates other proteins, including some ionic channels, changing their opening kinetics. As a result, PKA promotes the opening of calcium channels (Ca^2+^) L type and the closure of potassium channels (K^+^), leaving the neuron more susceptible to depolarization (Neve, 2009). Besides, stimulation of D_1_ receptors leads to a phosphorylation cascade that increases the α-amino-3-hydroxy-5-methyl-4-isoxazolepropionic acid (AMPA) (Xue et al., 2017) and N-methyl-d-aspartate (NMDA) receptors activity (Hallett, 2006) and, therefore, intensifies the excitatory responses of glutamatergic projections from the cortex (Figure 1C).

Conversely, the D_2_ receptor when stimulated presents an antagonistic effect to the D_1_ receptor. The D_2_ receptor works coupled to a G_α*i*_ protein, which inhibits adenylyl cyclase activity. For this reason, the cAMP intracellular accumulation is impaired. Consequently, there is no PKA activation, which remains unable to trigger the phosphorylation cascade that makes the neuron more predisposed to depolarization (Neve et al., 2004; Beaulieu and Gainetdinov, 2011). In addition to this pathway, there is a second pathway triggered by G_βγ_ subunits, where they activate phospholipase C (PLC) which acts by cleaving Phosphatidylinositol 4,5-bisphosphate (PIP2) into diacylglycerol (DAG) and inositol 1,2,4 trisphosphate (IP3). DAG acts by activating protein kinase C (PKC), while IP3 acts by releasing Ca^2+^ from the endoplasmic reticulum (Bonci and Hopf, 2005; Juza et al., 2023). Interestingly, in this scenario, the PLC pathway through D2 receptors can activate calcineurin which suppresses the opening of L-type Ca^2+^ channels, thus pushing the membrane potential away from the firing threshold and therefore the medium spiny neuron is less likely to depolarize (Hernández-López et al., 2000). This concept is essential to understand the direct and indirect pathways in the basal ganglia motor circuit (Figure 1D).

By this mechanism the D_2_ receptors stimulation suppress excitatory cortical afferences. The main inhibitory projections targets of striatal medium spiny neurons are GPe, GPi and SNr. Those that target the GPe have D_2_ receptors, and those that innervate GPi and SNr have D_1_ receptors (Lévesque et al., 2003). It is also important to mention the existing collateral connections between the axons of the D_1_ medium striatal spiny neurons with their D_2_ type homologs, generating feedback inhibition between these two types of striatal neurons (Plenz, 2003).

### 2.2. Intrinsic nuclei

Substantia nigra pars compacta (SNc) is a nucleus in the midbrain. Its neurons contain much melanin, conferring a dark coloration visible in fresh preparations. Due to this characteristic, it earned its name (Fabbri et al., 2017). Immunohistochemistry studies have shown that this nucleus also expresses the enzyme tyrosine hydroxylase, essential for dopamine synthesis (Cavalcanti et al., 2014, 2016; Rocha et al., 2022). In addition to striatal communications through dopaminergic projections, 3D imaging techniques have shown that SNc maintains connections with various cortical areas (Cacciola et al., 2016). In electrophysiological studies it was observed that the cortico-nigral connections are predominantly glutamatergic, and therefore excitatory (Meltzer et al., 1997). STN is another structure that sends excitatory inputs to the SNc. As seen by Shimo and Wichmann (2009), the STN of primates submitted to lesions presented lower rates of firing and dopamine release by SNc neurons than the control group. On the other hand, the SNr sends GABAergic projections to the adjoined SNc (Tepper and Lee, 2007).

The GPe, with GPi and putamen make up a structure known as lentiform nucleus. Although the lentiform nucleus subdivisions are topographically close and are composed of GABAergic projection neurons, they present communications with different areas, both afferent and efferent. Therefore, only the GPe is part of the intrinsic nuclei, since it receives projections from striatal medium spiny neurons and sends outputs to STN, GPi, SNr (Herrero et al., 2002), frontal cortex (Saunders et al., 2015), and also to striatal medium spiny neurons in a pathway known as arkypallidal pathway (Dunovan and Verstynen, 2016).

The STN also receives cholinergic projections from the peduncle pontine nucleus and glutamatergic ones from the cortex. The latter, in the basal ganglia motor circuit, is known as the hyperdirect pathway. The STN main synaptic targets are GPi, SNc and SNr. Since the STN is composed of neurons that predominantly use glutamate as a neurotransmitter, it acts by exciting these targets (Benarroch, 2008).

### 2.3. Efferent nuclei

The SNr and GPi have similar characteristics. Both are composed of GABAergic neurons that projects to the anterior ventral and lateral ventral nuclei of the thalamus, inhibiting its activity. In addition, both efferent nuclei receive inputs from the same regions: striatum, GPe and STN. However, the SNr maintains additional efferent connections with the superior colliculus and the SNc (Zhou and Lee, 2011). An important fact about the GPi is the different afferences according to their neuron’s location. While neurons at the center of the nucleus receive inhibitory synapses from the striatum and GPe, while neurons in peripheral regions receive inputs from STN (Purves et al., 2017).

### 2.4. Basal ganglia motor circuit

#### 2.4.1. Direct pathway

**FIGURE 2:**
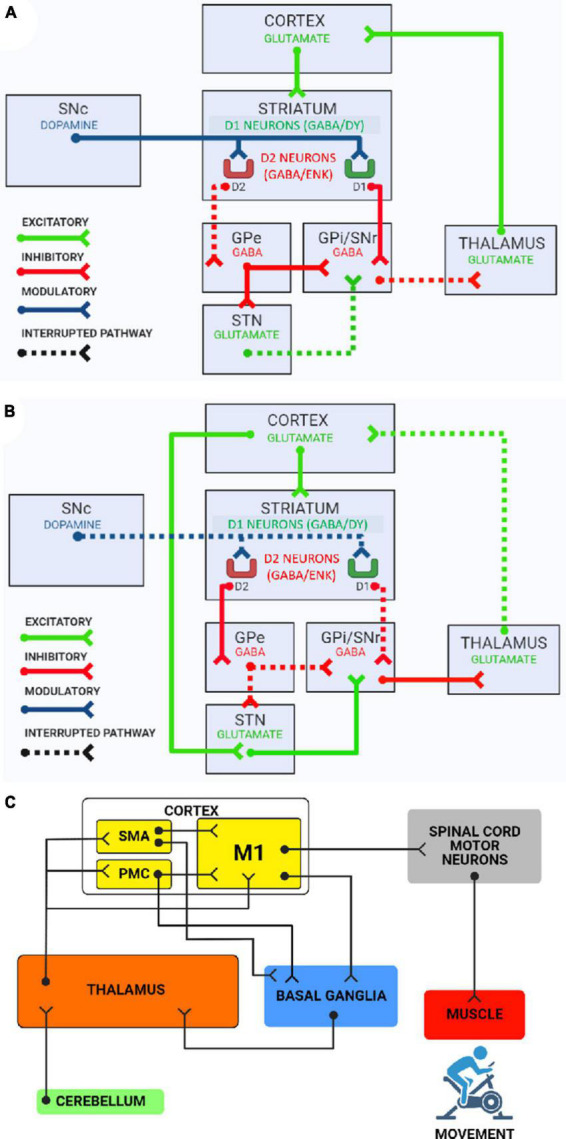
**(A)** The direct pathway of the basal ganglia motor circuit. Globus pallidus externus (GPe), globus pallidus internus (GPi), substantia nigra pars compacta (SNc), substantia nigra pars reticulata (SNr), and subthalamic nucleus (STN). The direct pathway results in tonic disinhibition of the VL nucleus of the thalamus, which in turn sends excitatory projections to the motor cortex, causing the activation of voluntary movement. **(B)** The indirect pathway of the basal ganglia motor circuit. The indirect pathway results in inhibition of the VL nucleus of the thalamus by GPe and SNr. This way VL nucleus of thalamus is not able to send excitatory projections to the motor cortex, causing the inhibition of undesired movements. **(C)** Overall circuit of voluntary movement. SMA, supplementary motor area; PMC, premotor cortex; M1, primary motor area. This scheme shows how the motor cortex, basal ganglia, thalamus, and cerebellum interact to promote precise voluntary movement. After the adjustment circuits involving these subcortical nuclei, neurons in the motor cortex excite α motor neurons in the spinal cord. Finally, α motor neurons stimulate muscle contraction, producing movement.

The motor circuit originates in the neocortex, which interacts with the striatum exciting this area with glutamate. Also, dopaminergic inputs from the SNc simultaneously regulate striatal activity. The striatum GABAergic neurons inhibit the GPi and the SNr. In parallel they are also inhibited by the GPe, which in the direct pathway is uninhibited, because the striatal neurons that project to this nucleus express D_2_ receptors and are inhibited by dopamine that comes from the SNc. In addition to inhibit the efferent nuclei directly, the GPe acts inhibiting the STN, which in turn cannot excite the GPi and the SNr. Therefore, the GPe inhibits doubly the efferent nuclei activity. Once inhibited, the GPi and the SNr are unable to inhibit the thalamus. Thus, thalamic neurons become disinhibited, able to excite the cortex (Graybiel, 2000), for this reason, the direct pathway acts in favor of voluntary movement (Figure 2A).

#### 2.4.2. Indirect pathway

In the indirect pathway, the striatum inhibits the GPe. In this way it cannot inhibit the STN, GPi and SNr. The STN, free of inhibition, receives excitatory cortical projections by the hyperdirect pathway (Nambu et al., 2002). When active, the STN excites GABAergic neurons of the efferent nuclei, which consequently inhibit the thalamus and render it incapable to excite the cortex (Calabresi et al., 2014). Therefore, indirect pathway acts to suppress unwanted movements, and in this way provide the most precise movement possible (Figure 2B).

#### 2.4.3. Hyperdirect pathway

In addition to the direct and indirect pathways, there is the hyperdirect pathway, formed by a monosynaptic axonal connection, which runs from the frontal cortex to the STN, using glutamate as neurotransmitter and is proposed to provide rapid inhibition to suppress motor action (Chen et al., 2020). The hyperdirect pathway evokes strong, short-latency excitatory responses in STN neurons that consequently excite GPi/SNr neurons, thus acting as a support for the indirect pathway to suppress unwanted movements (Nambu et al., 2002; Hasegawa et al., 2022).

#### 2.4.4. Dopaminergic modulation

The key to understand what determines the activity of both direct and indirect pathways are in the nigrostriatal dopaminergic pathway. Dopamine acts throughout the striatum and simultaneously binds on both D_1_ and D_2_ receptors. The neurons that hold D_1_ receptors projects to the efferent nuclei and control the direct pathway. On the other hand, neurons that have D_2_ receptors projects to GPe and command the indirect pathway. Through this mechanism dopamine inhibits the indirect pathway while activates the direct pathway (Gerfen and Surmeier, 2011). However, is important to know the nigrostriatal activity is determined by excitatory cortico-nigral projections (Gariano and Groves, 1988; Milardi et al., 2019) and inhibitory striato-nigral projections (Tepper and Lee, 2007). That being said, it is essential to make clear that the main role of SNc dopaminergic neurons in movement is to coordinate their initiation, but not having a significant participation in ongoing movements. Considering that just before a voluntary movement begins, there is a large increase in the firing of these neurons (Da Silva et al., 2018).

Moreover, it becomes more evident why the degeneration of dopaminergic neurons in the SNc caused by Parkinson’s disease inflicts immense difficulty to initiate voluntary movements in its carriers (Mazzoni et al., 2007). In this pathology the indirect pathway is always active, while the direct pathway is continuously inhibited (Obeso et al., 2000; Calabresi et al., 2014; Wichmann, 2019; Chakravarty et al., 2022). Complementarily, in scenarios where striatal dopamine depletion occurs, there is also disruption of the inhibition feedback between neurons in the direct pathway (D_1_) and neurons in the indirect pathway (D_2_). Thus, hyperactivity of the indirect pathway contributes to the underactivity of the direct pathway resulting in changes in motor patterns (Taverna et al., 2008; López-Huerta et al., 2013).

### 2.5. Basal ganglia and motor control

The basal ganglia role in motor control has been observed for a long time, especially in the studies of Marsden (1982), who first published the hypothesis that the basal ganglia are responsible for the execution of learned motor planning. Differently from what was thought when the classical models on the circuits of the basal ganglia were postulated, in which the direct and indirect pathways acted in an antagonistic manner, today it is known that these two pathways work in a synergistic and complementary manner (Jin and Costa, 2015; Klaus et al., 2019). As described by Cui et al. (2013), using electrophysiological records, it was observed that when mice started voluntary movement, both pathways were activated. Thus, being direct pathway neurons could select the desired motor program while indirect pathway neurons inhibit competing motor programs (Jin et al., 2014; Tecuapetla et al., 2014).

Pathological conditions such as Huntington’s and hemiballismus exemplify hyperkinetic disorders. Huntington’s disease is characterized by signs that include involuntary movements and motor impairments (Ross et al., 2014). This hyperkinesia originates from an autosomal dominant mutation in chromosome 4 as a pathological increase of the trinucleotide nucleotides cytosine, adenine and guanine (CAG) repetition (Bordelon, 2013). One of the repercussions is loss of striatal cells involved in the indirect pathway (Starr et al., 2008), thus favoring the direct pathway. Differently, in hemiballismus, a hyperkinetic disorder of intermittent, sudden and violent involuntary movements of the ipsilateral leg and arm, the causes are contralateral dysfunctions of the central nervous system (Grandas, 2011), such as STN injury (Nambu et al., 2002). In general, injuries in important regions of the indirect pathway or the direct pathway hyperactivity induces hyperkinesia.

On the other hand, lesions and disorders in important structures of the direct pathway cause hypokinesia, as seen in Parkinson’s disease, where the dopaminergic neuronal degeneration hinders the entire activation cascade for the direct pathway (Wichmann and DeLong, 1996; Höglinger et al., 2004). Therefore, the classic treatment is the administration of levodopa, a dopamine prodrug (Parkinson Study Group, 2004). Besides, it is possible to use deep brain stimulation of the STN, which seems to have an inhibitory effect on this nucleus (Milosevic et al., 2018), since the STN is an active component of the indirect pathway.

In addition, the basal ganglia and the cerebellum are reciprocally interconnected. As observed by Bostan and Strick (2010), in a paper using a viral tracer, the dentate nucleus of the cerebellum communicates with the ventral lateral (VL) nucleus of the thalamus, which in turn relays this information to the striatum. Secondly, STN neurons project into the pontine nuclei, and these into the cerebellar cortex. Together, basal ganglia and cerebellum are essential for the movement general circuit, as they modulate the activity of the premotor cortex, supplementary motor area and primary motor area, through a connection with the thalamus, which relays the information to the cortical areas (Thobois et al., 2000; Figure 2C).

A considerable part of patients with Parkinson’s disease suffers from resting tremor (Fishman, 2008). This condition can be explained by disorders in the interaction between basal ganglia and cerebellum. In this neuropathology, the STN presents over excitation due to the constant indirect pathway activation (Georgiades et al., 2019). And given that the STN influences the cerebellum activity (Bostan et al., 2010), it also becomes hyperactive (Yu et al., 2007). Taking into account that the GPi and dentate nucleus projections to the VL nucleus of the thalamus are related to movement control (Hintzen et al., 2018), the resting tremor occurs due to the cerebellum compensatory mechanisms, in order to counteract changes in the basal ganglia activity, which occurs in Parkinson’s disease, maintains the VL nucleus of the thalamus under strong inhibition (Wu and Hallett, 2013).

## 3. Conclusion

The basal ganglia role in movement control is crucial, and a proper knowledge about their structures, circuits, dopaminergic modulation, and dysfunctions is important. The present article sought to provide an integrated review addressing the main points of the subject in order to provide an updated overview about the issue.

## Author contributions

GR, MF, JC, and AB wrote the manuscript. GR, MF, and PM created and edited the figures. KP, RO, and IF reviewed updates on direct and indirect pathways. DA, LO, and FG assisted in the overall revision of the manuscript and formatting. GR, PM, and JC coordinated the manuscript construction process. All authors contributed to the article and approved the submitted version.
